# Genetic and Phenotypic Characterization of *Cryphonectria hypovirus 1* from Eurasian Georgia

**DOI:** 10.3390/v10120687

**Published:** 2018-12-03

**Authors:** Daniel Rigling, Nora Borst, Carolina Cornejo, Archil Supatashvili, Simone Prospero

**Affiliations:** 1 WSL Swiss Federal Research Institute, Zürcherstrasse 111, 8903 Birmensdorf, Switzerland; nora@breuert.eu (N.B.); carolina.cornejo@wsl.ch (C.C.); simone.prospero@wsl.ch (S.P.); 2 Vasil Gulisashvili Forestry Institute, Agricultural University of Georgia, 0186 Tbilisi, Georgia; archil.tbilisi@hotmail.com

**Keywords:** mycovirus, populations study, *Cryphonectria parasitica*, chestnut blight, *Castanea sativa*, biological control

## Abstract

*Cryphonectria hypovirus 1* (CHV-1) infects the chestnut blight fungus *Cryphonectria parasitica* and acts as a biological control agent against this harmful tree disease. In this study, we screened the recently characterized *C. parasitica* population in Eurasian Georgia for the presence of CHV-1. We found 62 CHV-1 infected *C. parasitica* isolates (9.3%) among a total of 664 isolates sampled in 14 locations across Georgia. The prevalence of CHV-1 at the different locations ranged from 0% in the eastern part of the country to 29% in the western part. Sequencing of two specific regions of the viral genome one each in ORFA and ORFB revealed a unique CHV-1 subtype in Georgia. This subtype has a recombinant pattern combining the ORFA region from the subtype F2 and the ORFB region from subtype D. All 62 viral strains belonged to this Georgian CHV-1 subtype (subtype G). The CHV-1 subtype G strongly reduced the parasitic growth of *C. parasitica* isolates from Georgia, with a more severe effect on the European genepool compared to the Georgian genepool. The CHV-1 subtype detected in Georgia provides a valuable candidate for biological control applications in the Caucasus region.

## 1. Introduction

Mycoviruses in plant pathogenic fungi have attracted interest because of their potential to be used as biological control agents against plant diseases [1]. One of the best studied examples is *Cryphonectria hypovirus 1* (CHV-1), which infects the chestnut blight fungus *Cryphonectria parasitica* [2,3]. This fungus is native to east Asia and has been introduced into North America and Europe [4]. After introduction of the pathogen, both continents experienced severe chestnut blight epidemics, which in eastern North America eliminated the American chestnut (*Castanea dentata*) as a dominant tree species [4]. The epidemic in Europe on the native European chestnut (*Castanea sativa*) initially took a very similar course, but after a certain time, recovery of many chestnut stands was observed [5]. This recovery has been attributed to hypovirulence, which refers to the viral disease caused by CHV-1 in *C. parasitica* [6]. The hypovirus induces a hypovirulent phenotype characterized by reduced virulence and sporulation capacity of the infected *C. parasitica* strains [7]. CHV-1 has an RNA genome, which contains two open reading frames (ORFs A and B). Both ORFs encode polyproteins, which are autocatalytically processed. The viral polymerase and helicase genes are located in the larger ORFB of the viral genome [2].

CHV-1 is located in the cytoplasm of the fungus and can be horizontally transmitted between fungal individuals through hyphal anastomosis [8]. From hypovirus-infected strains, CHV-1 may be vertically transmitted into asexual spores, which carry the hypovirus to new hosts [7,9]. The discovery of hypovirulence has led to the development of a biological control method against chestnut blight based on treatments of chestnut blight cankers with hypovirus-infected *C. parasitica* strains [10]. Hypovirulence is now a widespread phenomenon in many chestnut growing areas of Europe, either naturally or after biological control treatments [5,11,12].

Different CHV-1 subtypes or lineages have been identified in the European *C. parasitica* population [13,14]. Their distribution reflects the different introduction events of the fungal host. The Italian CHV-1 subtype (subtype I) is the most widespread and is linked to the introduction of *C. parasitica* into Italy in the 1930s [13]. Together with its fungal host, this subtype has spread across a large part of the chestnut growing areas in central, southern and southeastern Europe, including western Turkey [15,16,17]. Additional subtypes, designated F1, F2, and D/E, have mainly been found in western Europe (France, Spain and Germany) [14,18,19]. Interestingly, CHV-1 strains related to subtype F2 have also been identified in the eastern Black Sea region of Turkey [17]. Some subtypes apparently originated from recombination events [14,20]. The CHV-1 subtypes differ in their effects on the fungal host, with some variation within subtypes [7,21]. Strains of the Italian subtype typically have a mild effect on their hosts while subtypes F1 and F2 have a more severe impact [16,22,23].

Besides CHV-1, several other mycoviruses have been detected in *C. parasitica*, including not only additional members in the genus hypovirus (CHV-2, CHV-3, and CHV-4) but also viruses in other genera [24]. Some of these viruses (e.g., CHV-2, CHV-3) do also affect growth and fitness of *C. parasitica.* CHV-1, however, is most relevant for biological control of chestnut blight and to date the only mycovirus reported in *C. parasitica* from Europe.

The *C. parasitica* populations in the Caucasus region, i.e., in the most eastern distribution range of European chestnut, have only recently been genetically characterized. Microsatellite analysis indicated that most of the *C. parasitica* isolates from Georgia and Azerbaijan belong to a genepool, which largely differ from the genepools previously identified in Europe [25,26]. This finding points to an independent introduction of the fungus in the Caucasus area. However, *C. parasitica* isolates belonging to the European genepool were also identified in the western part of Georgia. These isolates were closely related to those previously described in neighboring Turkey, suggesting that they migrated from this country into Georgia.

The objectives of this study were to determine (1) the prevalence of CHV-1 in the *C. parasitica* population in Georgia, (2) the relatedness of the local CHV-1 strains to known CHV-1 subtypes from Europe, and (3) the phenotypic effects of the CHV-1 strains on fungal isolates from the European and Georgian genepool of *C. parasitica*.

## 2. Materials and Methods

### 2.1. Fungal Isolates

**Figure 1:**
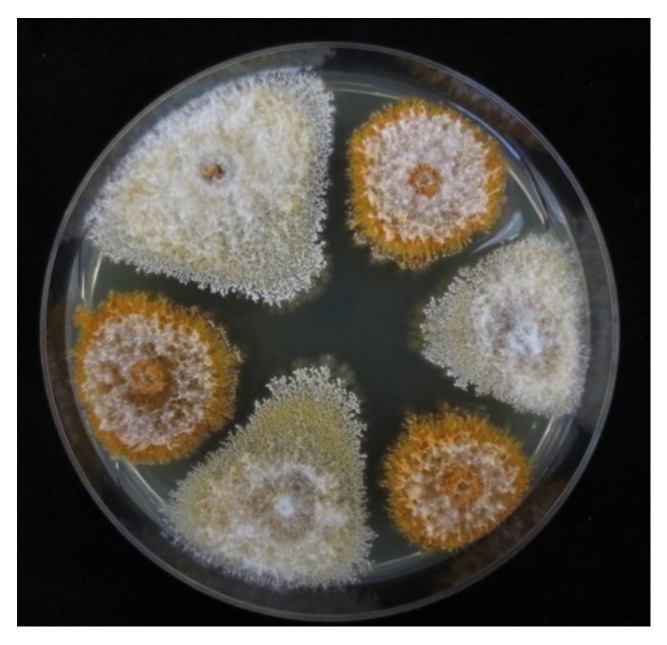
Culture morphology of CHV-1-free (orange) and CHV-1 infected (white) *C. parasitica* isolates from Georgia on PDA.

One set of isolates of *C. parasitica* was obtained from a previous study on the population structure of the chestnut blight fungus in Georgia [25]. Five additional populations (Shemoqmedi, Korbouli, Kumistavi, Satsable, Pshaveli) were sampled for this study in 2012 resulting in a total of 664 *C. parasitica* isolates (Table 1). All isolates were screened for the white culture morphology (Figure 1), which typically is associated with an infection by CHV-1 [7,16]. To assess the culture morphology, the isolates were grown on potato dextrose agar (PDA, Difco^TM^, Becton Dickinson, Sparks, MD, USA) as described previously [27]. All isolates exhibiting a white or intermediate (between white and orange) culture morphology were tested for the presence of CHV-1 by RT-PCR and sequencing.

### 2.2. RNA Extraction and RT-PCR

Isolates were grown on PDA plates overlaid with cellophane sheets for at least 6 days at room temperature in the dark. Mycelia reaching a diameter of approx. 6 cm were harvested, lyophilized, and ground to a fine powder in a mixer mill (MM 300 from Retsch, Haan, Germany). Total RNA was extracted from approx. 20 mg mycelial powder using the Norgen Plant/Fungi RNA Purification kit (Norgen Biotek Corp., Thorold, ON, Canada). Complementary DNA (cDNA) was synthesized from total RNA with random hexamer primers using the Maxima First Strand cDNA Synthesis kit from Fermentas (Thermo Fisher, Waltham, MA, USA).

### 2.3. Sequencing of CHV-1

Two different regions, one in ORFA and one in ORFB, were chosen for sequencing. The ORFA region corresponds to the positions 1471–2165 in the nucleotide sequence CHV-1/Euro7 [22] and was amplified using the primers described by Bryner, Rigling and Brunner [15]. The ORFB region was slightly modified after Feau et al. [14] and corresponds to the position 6264–6978. The forward primer ORFB-12aF (5′-AGACCTCAATCGGGTCTCCCT-3′) and the reverse primer ORFB-12aR (5′-TTCAACCACACGACGAGTTCG-3′) were used for PCR amplification. Sanger sequencing was performed using the same primers and the BigDye Terminator v3.1 Cycle Sequencing kit (Applied Biosystems, Foster City, CA, USA). Sequences were assembled and edited with DNADynamo (Blue Tractor Software Ltd., North Wales, UK). The GenBank accession numbers of all sequences are listed in Table S1.

### 2.4. Phylogenetic Analysis of CHV-1

Sequences were aligned using the web service Clustal Omega provided by the European Bioinformatics Institute (EMBL-EBI). Reference sequences for each CHV-1 subtype were generated as above or obtained from GenBank and are also listed in Table S1. Phylogenetic trees were reconstructed by maximum likelihood (ML) using the software PhyML 3.0 [28] on the ATGC platform (www.atgc-montpellier.fr). PhyML treats gaps systematically as unknown characters. Two random trees were used as starting trees corresponding to two random starts in order to decrease the chance of becoming trapped in a local maximum of the likelihood function. The starting trees were estimated using the BioNJ algorithm. Support for each internal branch of the phylogeny was calculated using the nonparametric bootstrap (B) option with 1000 pseudoreplicates, which estimates a phylogeny for each replicate. The online execution of PhyML includes the Smart Model Selection [29], which selects the model that best fit data under the Akaike Information Criterion and uses the selection direct in phylogenetic reconstruction.

Single locus trees were used to detect conflicting phylogenetic signal between the ORFA and ORFB region. For this purpose, clades of individual gene trees were examined for well-supported (≥70% of 1000 replicates) conflict between ML phylogenies. Following Vijaykrishna et al. [30], the change of the topological position of individuals was interpreted as a sign of reticulate evolutionary history. Unrooted phylogenies, including branch lengths and bootstrap support values, were graphically represented with SplitsTree4 (v.4.14.4; [31]).

### 2.5. Phenotypic Effects of the Georgian CHV-1 Subtype on C. parasitica

The effect of the Georgian CHV-1 subtype on the growth of the infected *C. parasitica* strains was assessed by conducting two different experiments in vitro similar as described by Bryner and Rigling [23]. The experiment on PDA plates was considered to reflect the saprophytic growth while that on dormant chestnut stems the parasitic growth of the fungus. For the experiments, 9–10 hypovirus-infected isolates with white culture morphology of each of the three Georgian genetic clusters of *C. parasitica* (CpGeo20 cluster, CpGeo75 cluster and CpGeo97 cluster) were randomly chosen among the 62 hypovirus-infected isolates identified in this study (Table S1). The CpGeo20 and CpGeo75 clusters represent the Georgian *C. parasitica* genepool, whereas the CpGeo97 cluster is related to isolates in vc type EU-1, which are present in Turkey and belong to the European genepool. To compare the effects of the Georgian and the Italian CHV-1 subtype, 10 Turkish isolates belonging to the vc type EU-1 and infected by the Italian CHV-1 subtype [21] were included in these experiments. From each virus-infected isolate, an isogenic virus-free isolate was obtained through single conidial isolation as described by Bryner and Rigling [21], resulting in 40 pairs of virus-infected and virus-free isolates. Virus-free isolates were distinguished from virus-infected isolates by their orange culture morphology (Figure 1).

#### 2.5.1. Growth on PDA Plates

For this experiment, three mycelial plugs (6 mm in diameter) originating from the growing margin of a 5-day-old pure culture on PDA of each hypovirus-infected isolate and its corresponding isogenic hypovirus-free isolate were placed each in the center of a PDA plate. The plates were sealed with Parafilm and incubated at 20 °C in a climate chamber under a 14L:10D photoperiod. Forty-eight hours after inoculation, the size of each culture was assessed the first time by measuring with a millimeter ruler the two cardinal diameters through two orthogonal axes previously drawn on the bottom of each plate. Measurements were repeated every 24 h for the next four days. Since the shape of the growing cultures were not perfect circles, the geometric mean diameter of an ellipse was calculated.

#### 2.5.2. Growth on Dormant Chestnut Stems

The same fungal isolates used for the previous experiment were inoculated on dormant chestnut (*Castanea sativa*) stems (5–10 cm in diameter, 50 cm in length) that were cut in December from three different sprout clusters in southern Switzerland (Ticino). Prior to inoculation, both ends of the stems were sealed with paraffin. For inoculation, four circular wounds (6 mm in diameter) reaching the cambium were made with a cork borer along the axis of each stem. Wounds were arranged 7 cm from each end of the stem and 12 cm apart from each other. Each wound was filled with one mycelial plug (6 mm in diameter) originating from the margin of a pure culture previously grown on PDA for 5 days in the dark. To prevent desiccation, wounds were subsequently sealed with masking tape. The inoculated stems were distributed horizontally on plastic grids located 5 cm above the bottom of 12 plastic containers (57 cm × 37 cm × 13 cm) so that each container hosted stems of all three sprout clusters. Hypovirus-free and hypovirus-infected isolates were assigned in pairs to chestnut stems. For each of the 40 pairs of isolates, three replicated inoculations were performed which were randomly distributed across containers and stems to avoid any influence of these factors on fungal growth. Containers were filled with demineralized water to a depth of 2 cm, closed with nontransparent lids and incubated at a constant temperature of 20 °C. After 28 days, the sizes of the lesions that developed at the inoculation points were determined using a millimeter ruler. Since these lesions had an elliptic shape, both the longitudinal and transversal diameters were measured. Thereafter, the geometric mean diameter was calculated.

### 2.6. Data Analysis

The CHV-1 virulence was quantified as the difference between the mean performances of the hypovirus-infected isolates and those of the corresponding isogenic hypovirus-free isolates and were given as a proportion (%) of the performances of the hypovirus-free isolate. A negative value indicated a higher performance of hypovirus-free isolates, whereas a positive value showed a higher performance of hypovirus-infected isolates. In the growth experiment on PDA, the performance of the *C. parasitica* isolates was estimated as the mean radial growth rate during the phase of linear growth [23]. To determine this phase, linear regression implemented in Microsoft Excel for Mac 2011 (Version 14.3.9) was performed (criterion: R^2^ > 0.98) on the geometric mean diameters of the cultures at 72, 96 and 120 h after inoculation. In the experiment with the dormant chestnut stems, the performance of the inoculated isolates corresponded to the geometric mean diameter of the lesion 28 days after inoculation.

All statistical analyses were performed in SPSS, version 22.0 (IBM^®^ SPSS^®^ Statistics). Mean performances of isolates were tested for significant differences (*p* < 0.05) by conducting a Tukey’s honestly significant difference test (Tukey’s HSD) in conjunction with a one-way analysis of variance (One Way ANOVA). This test identifies any difference between two means that is greater than the expected standard error. Model assumptions were checked with a Tukey-Anscombe plot and a Q-Q plot in R 3.1.2. Residuals were normally distributed and displayed constant error variances. To compare the effect of the Georgian and Italian CHV-1 subtypes on *C. parasitica* isolates from the European gene pool (CpGeo97 cluster from Georgia and EU-1 isolates from Turkey), an independent-samples *t*-test (*p* < 0.01) was performed. To test for a linear relationship between the virus effect on the fungal growth on dormant stems and on PDA, the Pearson’s correlation was calculated for all tested isolates.

## 3. Results

### 3.1. Prevalence and Genetic Characterization of CHV-1

Among the 664 *C. parasitica* isolates from Georgia, 62 isolates were tested positive for CHV-1 (Table 1). Of these 62 hypovirus-infected isolates, 58 exhibited a white and 4 an intermediate (between white and orange) culture morphology.

**Figure 2:**
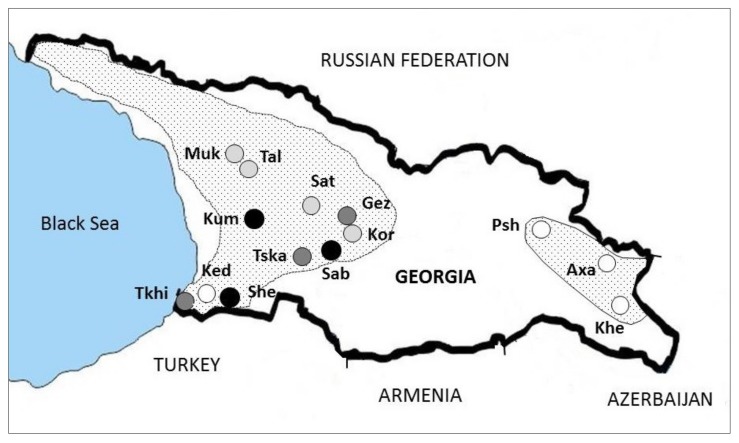
Prevalence and geographical distribution of CHV-1 in the *C. parasitica* populations in Georgia.

The prevalence of CHV-1 ranged from 0% to 29.8% with an average of 9.3%. CHV-1 was detected in three regions in western Georgia (Adjara, Imereti, Samegrelo-Zemo Svaneti), but not in the region Kakheti (populations of Axa, Khe and Psh), which is located in the eastern part of the country (Figure 2).

We obtained ORFA sequences from all 62 CHV-1 strains detected in Georgia. One strain failed to amplify the ORFB region resulting in 61 sequences for this region. In the ORFA region (695 nt), 100 sites were polymorphic, which corresponds to an average of 0.143 substitutions per site. Among the 62 sequences of the ORFA region, 52 haplotypes were identified. In the ORFB region (715 nt), 95 sites were polymorphic giving an average of 0.133 substitutions per site. A total of 54 haplotypes were found among the 61 sequences of the ORFB region. Pairwise identities among the ORFA sequences ranged from 97.3% to 100% and among the ORFB sequences from 96.6% to 100%.

### 3.2. Phylogenetic Analysis

**Figure 3:**
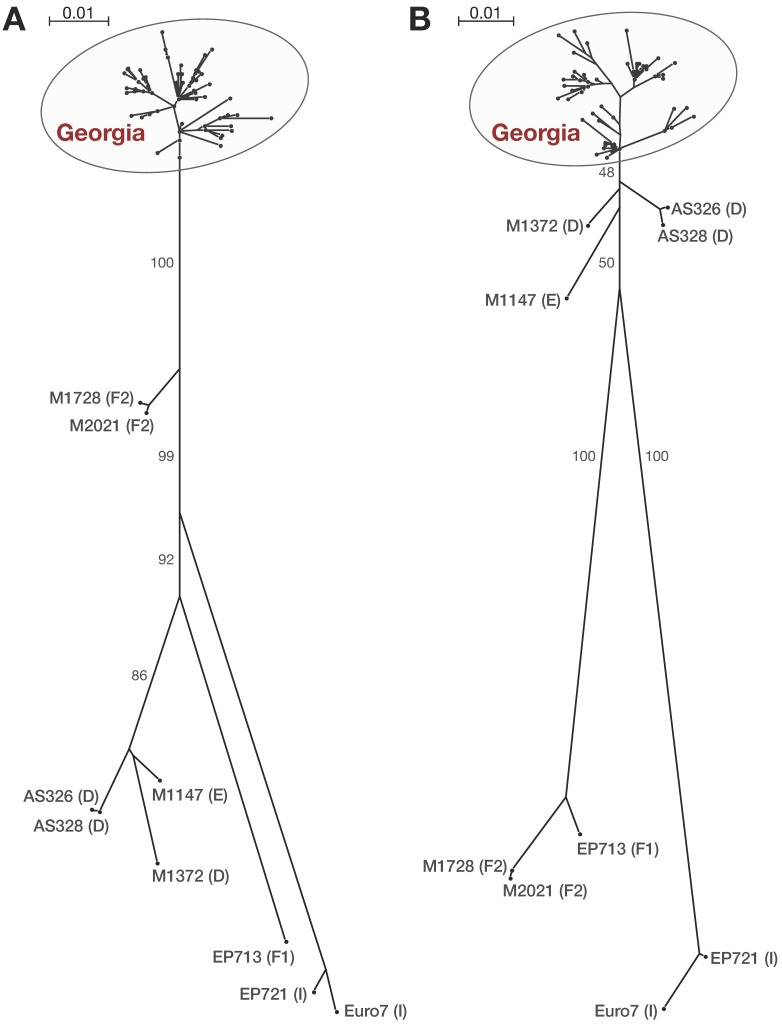
Individual gene trees of CHV-1 strains from Georgia and reference strains generated under maximum likelihood criterion (PhyML) using the GTR + gamma substitution model. (**A**) Gene tree of 71 ORFA sequences of CHV-1 (695 nt, 211 (30.4%) parsimony informative sites). (**B**) Gene tree of 70 ORFB sequences of CHV-1 (715 nt, 210 (29.4%) parsimony informative sites). Trees are unrooted. The scale bar represents the number of substitutions per site for a unit branch length. Numbers beside branches represent bootstraps support (%) of 1.000 bootstrap replicates. For the sake of simplicity, values of terminal branches are not shown and all CHV-1 strains from Georgia are indicated by a grey circle. Samples ID of the CHV-1 reference subtypes (D, E, F, F2 and I) are written beside nodes with the subtype denomination in brackets.

Including the reference sequences, the ORFA alignment contained 71 sequences and the ORFB alignment 70 sequences. Individual trees for both ORFs obtained from maximum likelihood analyses in PhyML are shown in the Figure 3. In both trees, all CHV-1 strains from Georgia clustered together in one group. In ORFA, this group was clearly separated from all other CHV-1 subtypes (D, E, F, F2, and I) with maximal support. A clear separation from the subtypes F1, F2, and I was also evident for ORFB. In this region, the CHV-1 strains from Georgia were closely related to the reference strains in subtypes D and E.

Comparison of both trees showed gene tree discordance, which occurs when phylogenies obtained from individual genes differ among themselves. This is clearly evident for the Georgian CHV-1 strains, which are most closely related to CHV-1 subtype F2 in ORFA but to subtype D in ORFB. Such contrasting tree topographies can be caused by genetic recombination that can occur in host cells co-infected with different virus strains [14]. Forcing incongruent phylogenies into a single species-tree analysis may result in discordant molecular convergence, which does not reflect any biological property of the sites where it occurs, being solely the result of the technical bias [32]. Therefore, the two ORF regions were not analyzed in a concatenated dataset. Since all CHV-1 strains from Georgia formed a distinct group in both ORFs, we propose a new subtype designation for this group, namely the Georgian CHV-1 subtype (CHV-1 subtype G). Subtype G is most closely related to the European lineage A2B2, i.e., A2 in ORFA and B2 in ORFB, described by Feau et al. [14].

### 3.3. Effect of the Georgian CHV-1 Subtype on Growth of C. parasitica

**Figure 4:**
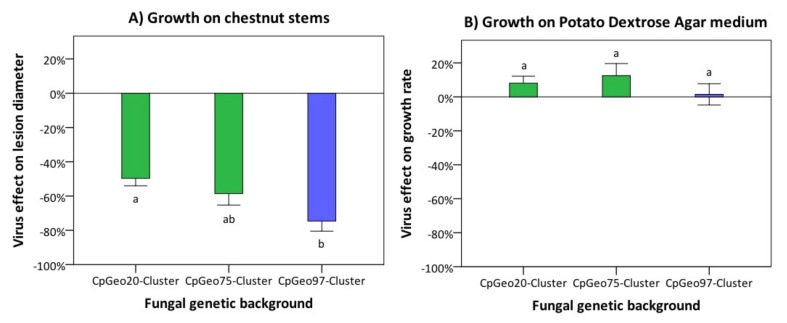
Effect of the Georgian CHV-1 subtype on the growth of its fungal host on dormant chestnut stems (**A**) and on PDA medium (**B**). For analyses, *C. parasitica* isolates were grouped according to their genetic background into the three genetic clusters CpGeo20 (*n* = 10), CpGeo75 (*n* = 10), and CpGeo97 (*n* = 9). Clusters CpGeo20 and CpGeo75 belong to the Georgian gene pool of *C. parasitica*, whereas cluster CpGeo97 to the European gene pool (for details on fungal genotypes see Prospero et al. [25]). The virus effect was quantified as the difference in performance between the hypovirus-infected and its isogenic hypovirus-free isolate as percentage of the performance of the hypovirus-free isolate. Error bars represent standard errors. Values with different letters differ significantly (*p* < 0.05).

The Georgian CHV-1 subtype had a different effect on the growth of its fungal host on dormant chestnut stems (parasitic growth) and on PDA medium (saprophytic growth) (Figure 4). On dormant chestnut stems, bark lesions induced by hypovirus-infected fungal isolates were significantly (*p* = 0.017) smaller than those caused by hypovirus-free isolates, independently from the genetic background of the fungal host. The mean hypovirus effect on lesion size across all isolates ranged from −22.8% (She27) to −92.7% (Tkhi48), with a mean value of −59.1% ± 3.8 (± SE; Table S2). The strongest hypovirus effect was observed on the *C. parasitica* cluster CpGeo97 (−74.6% ± 5.9) (Figure 4A). The isolates belonging to the clusters CpGeo20 and CpGeo75 experienced a milder effect (−48.3% ± 4.4 and −58.6% ± 6.7, respectively). Hypovirus-induced growth reduction differences were, however, only significant (*p* < 0.05) between cluster CpGeo20 and cluster CpGeo97. On PDA, the presence of the hypovirus did not significantly (*p* = 0.44) affect the growth rate of the infected *C. parasitica* isolates. A hypovirus infection resulted in a slight growth stimulation (+7.6% ± 3.4) and no significant differences were detected among the different genetic clusters of the fungus (cluster CpGeo97: +1.5% ± 6.2; cluster CpGeo20: +8.1% ± 4.2; cluster CpGeo75: +12.5% ± 7; Figure 4B).

### 3.4. Comparison of the Georgian and Italian CHV-1 Subtypes

**Figure 5:**
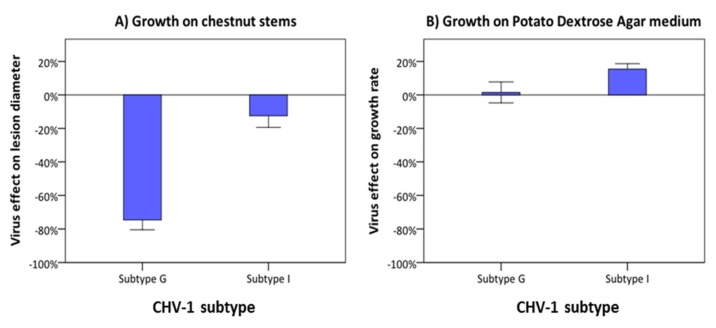
Effect of the Georgian (G) and the Italian (I) CHV-1 subtypes on the growth of *C. parasitica* isolates from the European gene pool on dormant chestnut stems (**A**) and on PDA (**B**). The virus effect was quantified as the difference in performance between the hypovirus-infected and its isogenic hypovirus-free isolate as percentage of the performance of the hypovirus-free isolate. Error bars represent the standard errors. Values with different letters differ significantly (*p* < 0.05).

The effect of the Georgian and Italian CHV-1 subtype on growth of the fungal host was compared in *C. parasitica* isolates belonging to the CpGeo97 cluster (European genepool). This cluster is represented by isolates from Georgia infected by the Georgian subtype (*n* = 9) and by isolates from Turkey infected by the Italian subtype (*n* = 10). Both subtypes reduced the fungal growth on dormant chestnut stem (Figure 5A), but the inhibitory effect of the Georgian subtype (−74.6% ± 5.9) was significantly higher (*p* < 0.01) than that of the Italian subtype (−12.5% ± 6.9). On PDA medium, both the Georgian and the Italian CHV-1 subtype slightly stimulated the growth of infected *C. parasitica* isolates (Figure 5B). The difference between the two subtypes was minor (Georgian subtype: 1.5% ± 6.2; Italian subtype: 15.5% ± 3.3) and statistically not significant (*p* > 0.05).

## 4. Discussion

As in Europe, chestnut forests in the Caucasus are suffering from the introduced chestnut blight fungus, *Cryphonectria parasitica*. In previous studies, a unique *C. parasitica* genepool was identified in this region, which is not related to the genepools occurring in Europe [25,26]. In the present study, we demonstrate that the Georgian *C. parasitica* population harbors a unique *Cryphonectria hypovirus* population. All hypovirus strains detected formed distinct phylogenetic groups in both regions analyzed. Following the CHV-1 subtype designation used for European hypoviruses ([13,33], we propose the new hypovirus group to be named the Georgian CHV-1 subtype (subtype G).

In relation to the previously described CHV-1 subtypes from Europe, conflicting phylogenetic signals between the ORFA and ORB regions were obtained for the Georgian CHV-1 strains. In ORFA the analyzed strains were most closely related to the CHV-1 subtype F2 and in ORFB to the subtypes D/E. Finding gene tree discordance in viral populations is not surprising, as phylogenetic incongruence has been often observed within viral strains [34,35]. This phenomenon may be the consequence of viral recombination, which occurs when two different virus strains co-infect a single cell. The viral RNA-dependent-RNA-polymerase can dissociate from the first genome and continue replication by using the second distinct genome as template [30,36]. The result is a novel mosaic-like genome with regions from different sources. Recombination events have been previously described in CHV-1 and very likely have contributed to the evolution of CHV-1 subtypes in Europe [14,20]. The study of Feau et al. [14] showed that two recombinant CHV-1 lineages (subtype F1 and I) were more frequent than the putative parental strains suggesting an increased invasiveness of the recombinants.

Mlinarec et al. [20] recently provided full genome sequences of all European subtypes including one CHV-1 strain from Georgia. Recombinant analysis suggested that the strain from Georgia has a recombinant pattern between CHV-1 subtype I (minor parent) and the subtypes F1, D, E (major parent). Our study indicates that these putative parental strains do not occur in Georgia and suggests that the recombination event took place outside of this area. Western Europe (France, Spain) could be the center of origin of the Georgian subtype since all putative parental CHV-1 subtypes have been found in that area [14,19,37]. However, there is no indication of any relationship between the *C. parasitica* population in western Europe and Georgia [25]. To our knowledge there are also no records for any movement of chestnut plant material between the two areas, which could have spread hypovirus-infected *C. parasitica* isolates. Alternatively, and more likely, the Georgian subtype has evolved somewhere in Asia, i.e., in the native range of *C. parasitica* and CHV-1. *C. parasitica* isolates harboring such recombinant CHV-1 strains were then introduced into Georgia and founded both the fungal and virus population in this country. To our knowledge artificial application of hypovirus-infected *C. parasitica* strains to control chestnut blight have never been performed in the Caucasus region including eastern Turkey. Thus, the unique CHV-1 subtype identified in Georgia was very likely introduced there together with its fungal host from Asia. The recent finding of related CHV-1 strains in Eastern Turkey [17] suggests that CHV-1 subtype G is slowly migrating westwards.

Our study revealed distinct hypovirus x fungus interactions for parasitic growth of the fungus on dormant chestnut stems, but not for saprophytic growth on PDA plates. On chestnut stems, the Georgian CHV-1 subtype was found to have a different effect on the growth of *C. parasitica* depending on the genetic background of the fungal host strains. This was most evident when comparing the Georgian CpGeo20 cluster with the European CpGeo97 cluster, which experienced a significant greater growth reduction than the former cluster. It has been hypothesized that mycoviruses will adapt to its fungal hosts and evolve towards milder strains, in order not to severely reduce the fitness of the hosts [21,38]. This hypothesis is based on the fact that most mycoviruses, including CHV-1, do not have an extracellular phase and fully depend on their hosts for survival and spread [1,2]. Along this line, our results suggest that the Georgian CHV-1 subtype is more adapted to the Georgian *C. parasitica* genepool than to the European genepool. This assumption is consistent with the scenario that the Georgian CHV-1 subtype has been present for a longer time in the Georgian genepool and only recently infected the European genepool that had migrated from Turkey into Georgia. Similarly, the Georgian CHV-1 subtype had a more severe effect on the European genepool than the Italian subtype, which has shared a long invasion history with the European *C. parasitica* population [15].

Hypovirus-mediated biological control of chestnut blight has been successful in many areas in Europe, either naturally or after artificial application [5]. The basis of biocontrol is provided by the ability of CHV-1 to reduce the parasitic growth (virulence) of infected *C. parasitica* isolates. An additional important characteristic of hypovirulence is the transmissibility of CHV-1 among fungal individuals. This horizontal hypovirus transmission is controlled by the vegetative incompatibility system in *C. parasitica* [8]. If barriers imposed by this system are not too high, CHV-1 is able to infect a large proportion of a fungal population [39]. In Europe, where hypovirulence is successful it is not uncommon to find a hypovirus prevalence of more than 50% [3]. In Georgia, we found three locations with a CHV-1 prevalence between 20% and 30%. In most other locations, the prevalence was much lower with zero detection in the eastern part of the country. Even at a relatively low prevalence, CHV-1 might still mitigate the chestnut blight severity in Georgia at least to a certain degree and particularly in the western part of the country. Our phenotypic analysis supports this assumption by demonstrating that the Georgian CHV-1 subtype strongly reduces the parasitic growth of all three genetic clusters of *C. parasitica* identified in Georgia. It is interesting to note that Georgian CHV-1 subtype did not reduce the saprophytic growth of *C. parasitica* as assessed on PDA plates. This is a typical characteristic of many CHV-1 strains, particularly those of the Italian subtype [16]. The saprophytic ability of hypovirus-infected *C. parasitica* to growth and sporulate on dead chestnut stems is thought to be important for the success of natural hypovirulence in Europe [9] and probably also plays a role in the natural chestnut forests in Georgia.

In eastern Georgia, where CHV-1 was not found, biological control applications are highly feasible using CHV-1 strains from the western part of the country. The genetic diversity of *C. parasitica* is low in the eastern part with a single dominant genotype (CpGeo20) present [25]. Therefore, there are almost no vegetative incompatibility (*vic*) barriers, which should favor the spread of an artificially applied hypovirus. Hypovirulence application in the western part of the country is more problematic since the local *C. parasitica* population is genetically highly diverse as determined by microsatellite markers [25]. Vegetative incompatibility so far has not been studied in Georgia. Studies in other areas, however, showed that *vic* genotype diversity is highly correlated with microsatellite diversity [26,40,41]. Therefore, we can assume that *vic* barriers are strong in western Georgia and are probably responsible for the low prevalence of CHV-1 in local *C. parasitica* populations.

In our study, we focused on CHV-1, which is the most efficient biological control agent against chestnut blight and to date the only mycovirus reported in *C. parasitica* from Europe. In North America and Asia, several other mycoviruses have been found in the chestnut blight fungus [24]. It is likely that *Cryphonectria* mycoviruses other than CHV-1 also occur in Europa including Georgia, but screening for the presence of such viruses remains to be done.

## 5. Conclusions

Our study reveals the occurrence of a unique CHV-1 subtype in the *C. parasitica* population in Georgia. This finding is consistent with a previous study showing that there is a unique fungal genepool in Georgia, which is not related to other *C. parasitica* genepools in Europe. The Georgian CHV-1 subtype belongs to the more severe hypoviruses as it strongly reduced the parasitic growth of *C. parasitica*. Although this CHV-1 subtype overall has a strong phenotypic effect on *C. parasitica*, it seems to be more adapted to the Georgian than to the European genepool of *C. parasitica*. This suggests a longer interaction history with the Georgian than with the European genepool, which probably had migrated only recently from Turkey into Georgia. The CHV-1 subtype in Georgia provides a valuable biological control agent for hypovirulence applications in the Caucasus region. 

## Figures and Tables

**Table 1:** Prevalence of CHV-1 in the *Cryphonectria parasitica* populations sampled in Georgia.

Population ^1^	Region	N C.p. ^2^	N CHV-1 ^3^	% CHV-1
Keda (Ked)	Adjara	48	0	0
Shemoqmedi (She)	Adjara	47	14	29.8
Tkilnari (Tkhi)	Adjara	48	5	10.4
Gezruli (Gez)	Imereti	45	8	17.8
Korbouli (Kor)	Imereti	44	2	4.5
Kumistavi (Kum)	Imereti	50	12	24.0
Satsable (Sab)	Imereti	47	11	23.4
Satsire (Sat)	Imereti	71	3	4.2
Tskalthashua (Tska)	Imereti	46	4	8.7
Mukhuri (Muk)	Samegrelo-Zemo Svaneti	48	1	2.1
Taleri (Tal)	Samegrelo-Zemo Svaneti	43	2	4.7
Axalsopeli (Axa)	Kakheti	29	0	0
Khecili (Khe)	Kakheti	49	0	0
Pshaveli (Psh)	Kakheti	49	0	0
Total		664	62	9.3

^1^ Abbreviation of each population in brackets. ^2^ Number of *C. parasitica* isolates per population. ^3^ Number of *C. parasitica* isolates tested positive for CHV-1.

## Supplementary Materials

The following are available online at http://www.mdpi.com/1999-4915/10/12/687/s1, Table S1: List of CHV-1 strains used in this study and GenBank accession numbers of the CHV-1 sequences, Table S2: Effect of the Georgian CHV-1 subtype on the growth of the infected *Cryphonectria parasitica* isolates on PDA and on dormant chestnut stems.

## References

[1] Ghabrial S.A., Suzuki N. (2009). Viruses of plant pathogenic fungi. Annu. Rev. Phytopathol..

[2] Nuss D.L. (2005). Hypovirulence: Mycoviruses at the fungal-plant interface. Nat. Rev. Microbiol..

[3] Rigling D., Prospero S. (2018). *Cryphonectria parasitica*, the causal agent of chestnut blight: Invasion history, population biology and disease control. Mol. Plant Pathol..

[4] Anagnostakis S.L. (1987). Chestnut blight—The classical problem of an introduced pathogen. Mycologia.

[5] Heiniger U., Rigling D. (1994). Biological control of chestnut blight in Europe. Annu. Rev. Phytopathol..

[6] Choi G.H., Nuss D.L. (1992). Hypovirulence of chestnut blight fungus conferred by an infectious viral cDNA. Science.

[7] Peever T.L., Liu Y.C., Cortesi P., Milgroom M.G. (2000). Variation in tolerance and virulence in the chestnut blight fungus-hypovirus interaction. Appl. Environ. Microb..

[8] Cortesi P., McCulloch C.E., Song H.Y., Lin H.Q., Milgroom M.G. (2001). Genetic control of horizontal virus transmission in the chestnut blight fungus, *Cryphonectria parasitica*. Genetics.

[9] Prospero S., Conedera M., Heiniger U., Rigling D. (2006). Saprophytic activity and sporulation of *Cryphonectria parasitica* on dead chestnut wood in forests with naturally established hypovirulence. Phytopathology.

[10] Grente M.J. (1965). Les formes hypovirulentes d’*Endothia parasitica* et les espoirs de lutte contre le chancre du châtaignier. Académie D’agriculture de France.

[11] Diamandis S. (2018). Management of chestnut blight in Greece using hypovirulence and silvicultural interventions. Forests.

[12] Bryner S.F., Prospero S., Rigling D. (2014). Dynamics of Cryphonectria hypovirus infection in chestnut blight cankers. Phytopathology.

[13] Gobbin D., Hoegger P.J., Heiniger U., Rigling D. (2003). Sequence variation and evolution of *Cryphonectria hypovirus 1* (CHV-1) in Europe. Virus Res..

[14] Feau N., Dutech C., Brusini J., Rigling D., Robin C. (2014). Multiple introductions and recombination in *Cryphonectria hypovirus 1*: Perspective for a sustainable biological control of chestnut blight. Evol. Appl..

[15] Bryner S.F., Rigling D., Brunner P.C. (2012). Invasion history and demographic pattern of *Cryphonectria hypovirus 1* across European populations of the chestnut blight fungus. Ecol. Evol..

[16] Robin C., Lanz S., Soutrenon A., Rigling D. (2010). Dominance of natural over released biological control agents of the chestnut blight fungus *Cryphonectria parasitica* in South-Eastern France is associated with fitness-related traits. Biol. Control.

[17] Akilli S., Serce C.U., Katircioglu Y.Z., Maden S., Rigling D. (2013). Characterization of hypovirulent isolates of the chestnut blight fungus, *Cryphonectria parasitica* from the Marmara and Black Sea regions of Turkey. Eur. J. Plant Pathol..

[18] Peters F.S., Busskamp J., Prospero S., Rigling D., Metzler B. (2014). Genetic diversification of the chestnut blight fungus *Cryphonectria parasitica* and its associated hypovirus in Germany. Fungal Biol..

[19] Trapiello E., Rigling D., Gonzalez A.J. (2017). Occurrence of hypovirus-infected *Cryphonectria parasitica* isolates in northern Spain: An encouraging situation for biological control of chestnut blight in Asturian forests. Eur. J. Plant Pathol..

[20] Mlinarec J., Nuskern L., Jezic M., Rigling D., Curkovic-Perica M. (2018). Molecular evolution and invasion pattern of *Cryphonectria hypovirus 1* in Europe: Mutation rate, and selection pressure differ between genome domains. Virology.

[21] Bryner S.F., Rigling D. (2012). Hypovirus virulence and vegetative incompatibility in populations of the chestnut blight fungus. Phytopathology.

[22] Chen B.S., Nuss D.L. (1999). Infectious cDNA clone of hypovirus CHV1-Euro7: A comparative virology approach to investigate virus-mediated hypovirulence of the chestnut blight fungus *Cryphonectria parasitica*. J. Virol..

[23] Bryner S.F., Rigling D. (2011). Temperature-dependent genotype-by-genotype interaction between a pathogenic fungus and its hyperparasitic virus. Am. Nat..

[24] Hillman B.I., Suzuki N. (2004). Viruses of the chestnut blight fungus, *Cryphonectria parasitica*. Adv. Virus Res..

[25] Prospero S., Lutz A., Tavadze B., Supatashvili A., Rigling D. (2013). Discovery of a new gene pool and a high genetic diversity of the chestnut blight fungus *Cryphonectria parasitica* in Caucasian Georgia. Infect. Genet. Evol..

[26] Aghayeva D.N., Rigling D., Prospero S. (2017). Low genetic diversity but frequent sexual reproduction of the chestnut blight fungus *Cryphonectria parasitica* in Azerbaijan. For. Pathol..

[27] Bissegger M., Rigling D., Heiniger U. (1997). Population structure and disease development of *Cryphonectria parasitica* in European chestnut forests in the presence of natural hypovirulence. Phytopathology.

[28] Guindon S., Dufayard J.F., Lefort V., Anisimova M., Hordijk W., Gascuel O. (2010). New algorithms and methods to estimate maximum-likelihood phylogenies: Assessing the performance of PhyML 3.0. Syst. Biol..

[29] Lefort V., Longueville J.E., Gascuel O. (2017). SMS: Smart model selection in PhyML. Mol. Biol. Evol..

[30] Vijaykrishna D., Mukerji R., Smith G.J.D. (2015). RNA virus reassortment: An evolutionary mechanism for host jumps and immune evasion. PLoS Pathog..

[31] Huson D.H., Bryant D. (2006). Application of phylogenetic networks in evolutionary studies. Mol. Biol. Evol..

[32] Mendes F.K., Hahn Y., Hahn M.W. (2016). Gene tree discordance can generate patterns of diminishing convergence over time. Mol. Biol. Evol..

[33] Allemann C., Hoegger P., Heiniger U., Rigling D. (1999). Genetic variation of *Cryphonectria* hypoviruses (CHV1) in Europe, assessed using restriction fragment length polymorphism (RFLP) markers. Mol. Ecol..

[34] Wani S.A., Sahu A.R., Mishra B.P., Kumar A., Priya G.B., Padhy A., Sahoo A.P., Tiwari A.K., Gandham R.K., Singh R.K. (2015). Whole genome sequence analysis of viruses; moving beyond single/partial gene based phylogenies in context of epidemiology and genetic evolution. Adv. Anim. Vet. Sci..

[35] Kostaki E.G., Karamitros T., Stefanou G., Mamais I., Angelis K., Hatzakis A., Kramvis A., Paraskevis D. (2018). Unravelling the history of hepatitis B virus genotypes A and D infection using a full-genome phylogenetic and phylogeographic approach. eLife.

[36] Simon-Loriere E., Holmes E.C. (2011). Why do RNA viruses recombine?. Nat. Rev. Microbiol..

[37] Zamora P., Martin A.B., Rigling D., Diez J.J. (2012). Diversity of *Cryphonectria parasitica* in western Spain and identification of hypovirus-infected isolates. For. Pathol..

[38] Bryner S.F., Rigling D. (2012). Virulence not only costs but also benefits the transmission of a fungal virus. Evolution.

[39] Brusini J., Robin C. (2013). Mycovirus transmission revisited by in situ pairings of vegetatively incompatible isolates of *Cryphonectria parasitica*. J. Virol. Methods.

[40] Jezic M., Krstin L., Rigling D., Curkovic-Perica M. (2012). High diversity in populations of the introduced plant pathogen, *Cryphonectria parasitica*, due to encounters between genetically divergent genotypes. Mol. Ecol..

[41] Prospero S., Rigling D. (2012). Invasion genetics of the chestnut blight fungus *Cryphonectria parasitica* in Switzerland. Phytopathology.

