# Supplementary material for: Genetic and Phenotypic Characterization of Cryphonectria hypovirus 1 from Eurasian Georgia
Source: Viruses. 2018 Dec 3;10(12):687. doi: 10.3390/v10120687 (PMC6315935; doi:10.3390/v10120687)
Supplement: Supplementary file 1 [file viruses-10-00687-s001.zip › Tables S1 and S2/Table S2.docx]

**Table S2**. Effect of the Georgian and Italian CHV-1 subtype on the growth of the infected *Cryphonectria parasitica* isolates on PDA and on dormant chestnut stems.

| **Isolate** | **Origin** | **Genetic background^1^** | **CHV-1 subtype** | **Growth rate on PDA^2^** | | | **Lesion size on chestnut stems^3^** | | |
| --- | --- | --- | --- | --- | --- | --- | --- | --- | --- |
|  |  |  |  | **-CHV-1 (mm/day)** | **+CHV-1 (mm/day)** | **V_eff_ (%)** | **-CHV-1 (mm)** | **+CHV-1 (mm)** | **V_eff_ (%)** |
| Gez 45/2 | Georgia | CpGeo20 | Georgian | 4.8 | 5.6 | 18.5 | 25.3 | 11.4 | -54.9 |
| Kor 39 | Georgia | CpGeo20 | Georgian | 5.4 | 5.2 | -3.8 | 30.6 | 19.7 | -35.6 |
| Muk 37b | Georgia | CpGeo20 | Georgian | 4.6 | 5.6 | 21.7 | 34.2 | 17.1 | -49.9 |
| Sab 3 | Georgia | CpGeo20 | Georgian | 5.1 | 4.8 | -6.9 | 36.0 | 12.8 | -64.3 |
| Sab 4 | Georgia | CpGeo20 | Georgian | 4.8 | 4.7 | -1.7 | 47.6 | 19.2 | -59.6 |
| She 9 | Georgia | CpGeo20 | Georgian | 5.0 | 4.3 | -13.0 | 36.9 | 22.8 | -38.2 |
| Sab 16 | Georgia | CpGeo20 | Georgian | 4.6 | 5.6 | 21.0 | 25.9 | 8.5 | -67.2 |
| She 27 | Georgia | CpGeo20 | Georgian | 4.8 | 5.7 | 20.5 | 30.1 | 23.2 | -22.8 |
| Tska 17b | Georgia | CpGeo20 | Georgian | 4.9 | 5.6 | 14.9 | 48.6 | 24.2 | -50.2 |
| Tska 28 | Georgia | CpGeo20 | Georgian | 5.2 | 5.7 | 9.6 | 35.5 | 21.3 | -40.0 |
| Kor 26 | Georgia | CpGeo75 | Georgian | 5.6 | 6.0 | 6.8 | 41.0 | 19.8 | -51.8 |
| Kum 19 | Georgia | CpGeo75 | Georgian | 4.9 | 6.8 | 38.2 | 44.8 | 20.1 | -55.1 |
| Kum 3 | Georgia | CpGeo75 | Georgian | 5.3 | 7.1 | 34.5 | 27.2 | 18.1 | -33.5 |
| Kum 39 | Georgia | CpGeo75 | Georgian | 5.1 | 6.4 | 25.4 | 40.4 | 25.0 | -38.1 |
| Kum 40 | Georgia | CpGeo75 | Georgian | 5.6 | 6.7 | 20.7 | 40.6 | 27.5 | -32.1 |
| Sab 50 | Georgia | CpGeo75 | Georgian | 3.3 | 3.6 | 8.4 | 34.3 | 8.5 | -75.2 |
| Sat 20 | Georgia | CpGeo75 | Georgian | 5.7 | 6.0 | 5.1 | 29.0 | 7.2 | -75.0 |
| She 12 | Georgia | CpGeo75 | Georgian | 4.6 | 2.7 | -41.3 | 32.3 | 5.0 | -84.6 |
| She 31 | Georgia | CpGeo75 | Georgian | 5.3 | 5.5 | 5.1 | 31.1 | 3.3 | -89.3 |
| She 46 | Georgia | CpGeo75 | Georgian | 5.1 | 6.2 | 22.0 | 34.9 | 17.2 | -50.8 |
| Gez 31 | Georgia | CpGeo97 | Georgian | 4.6 | 3.0 | -34.1 | 25.3 | 4.6 | -81.9 |
| Kum 7 | Georgia | CpGeo97 | Georgian | 5.1 | 6.0 | 17.2 | 38.8 | 22.0 | -43.4 |
| Kum 16 | Georgia | CpGeo97 | Georgian | 4.9 | 5.4 | 11.1 | 27.4 | 4.2 | -84.5 |
| Kum 37 | Georgia | CpGeo97 | Georgian | 5.0 | 6.2 | 22.8 | 30.7 | 8.7 | -71.7 |
| She 24 | Georgia | CpGeo97 | Georgian | 4.8 | 4.8 | 1.2 | 33.9 | 3.2 | -90.7 |
| She 30 | Georgia | CpGeo97 | Georgian | 5.4 | 5.4 | 0.3 | 41.6 | 16.0 | -61.5 |
| She 37a | Georgia | CpGeo97 | Georgian | 5.3 | 5.5 | 4.5 | 30.5 | 3.2 | -89.4 |
| Tkhi 39a | Georgia | CpGeo97 | Georgian | 5.8 | 6.7 | 14.7 | 32.1 | 14.2 | -55.8 |
| Tkhi 48 | Georgia | CpGeo97 | Georgian | 5.7 | 4.5 | -21.8 | 38.0 | 2.8 | -92.7 |
| Bu1 | Turkey | EU-1 | Italian | 4.7 | 4.5 | -4.0 | 31.6 | 27.8 | -12.1 |
| Bu17 | Turkey | EU-1 | Italian | 4.8 | 5.7 | 19.1 | 34.5 | 35.0 | 1.3 |
| Bu33 | Turkey | EU-1 | Italian | 4.5 | 5.4 | 19.7 | 37.2 | 36.7 | -1.4 |
| Bu44 | Turkey | EU-1 | Italian | 5.1 | 5.5 | 7.8 | 35.9 | 28.0 | -21.9 |
| Bu97 | Turkey | EU-1 | Italian | 4.9 | 5.6 | 13.7 | 35.1 | 36.5 | 4.1 |
| Ya14 | Turkey | EU-1 | Italian | 3.8 | 3.9 | 2.8 | 37.3 | 11.7 | -68.6 |
| Ya28 | Turkey | EU-1 | Italian | 4.5 | 5.4 | 20.8 | 39.1 | 37.9 | -2.9 |
| Ya56 | Turkey | EU-1 | Italian | 4.7 | 5.9 | 23.7 | 25.6 | 19.7 | -23.0 |
| Ya79 | Turkey | EU-1 | Italian | 4.7 | 5.8 | 21.8 | 28.5 | 28.0 | -1.9 |
| Ya97 | Turkey | EU-1 | Italian | 5.0 | 6.4 | 28.9 | 35.4 | 36.0 | 1.7 |

^1^Genetic cluster of the CHV-1 infected *C. parasitica* isolates according to Prospero et al. (2013).

^2^Growth rate of a virus-infected isolate (+CHV-1)) and its isogenic virus-free isolate (-CHV-1) on PDA medium and effect of the virus (V_eff_) as the difference in performance between the virus-infected and the virus-free isolate as percentage of the performance of the virus-free isolate.

^3^Size (geometric mean diameter) of the lesion induced by a virus-infected isolate (+CHV-1) and its isogenic virus-free isolate (-CHV-1) on dormant chestnut stems (28 days after inoculation) and effect of the virus (V_eff_) as the difference in performance between the virus-infected and the virus-free isolate as percentage of the performance of the virus-free isolate.
