# Supplementary material for: Genetic and Phenotypic Characterization of Cryphonectria hypovirus 1 from Eurasian Georgia
Source: Viruses. 2018 Dec 3;10(12):687. doi: 10.3390/v10120687 (PMC6315935; doi:10.3390/v10120687)
Supplement: Supplementary file 1 [file viruses-10-00687-s001.zip › Tables S1 and S2/Table S1 .docx]

**Table S1.** List of CHV-1 strains used in this study and Genbank accession numbers of the CHV-1 sequences

| **CHV1 strain** | **Location** | **Country** | **Collection date** | **Accession numbers of ORFA region** | **Accession numbers of ORFB region** | **Reference** |
| --- | --- | --- | --- | --- | --- | --- |
| **CHV1 strains from Georgia** | | |  |  |  |  |
| Gez9 | Gezruli | Georgia | 2010 | MK031045 | MK031107 | This study |
| Gez25 | Gezruli | Georgia | 2010 | MK031046 | MK031108 | This study |
| Gez26 | Gezruli | Georgia | 2010 | MK031047 | MK031109 | This study |
| Gez30 | Gezruli | Georgia | 2010 | MK031048 | MK031110 | This study |
| Gez31 | Gezruli | Georgia | 2010 | MK031049 | MK031111 | This study |
| Gez32 | Gezruli | Georgia | 2010 | MK031050 | MK031112 | This study |
| Gez45_2 | Gezruli | Georgia | 2010 | MK031051 | MK031113 | This study |
| Gez48 | Gezruli | Georgia | 2010 | MK031052 | MK031114 | This study |
| Kor26 | Korbouli | Georgia | 2012 | MK031053 | MK031115 | This study |
| Kor39 | Korbouli | Georgia | 2012 | MK031054 | MK031116 | This study |
| Kum3 | Kumistavi | Georgia | 2012 | MK031055 | MK031117 | This study |
| Kum7 | Kumistavi | Georgia | 2012 | MK031056 | MK031118 | This study |
| Kum9 | Kumistavi | Georgia | 2012 | MK031057 | MK031119 | This study |
| Kum15 | Kumistavi | Georgia | 2012 | MK031058 | MK031120 | This study |
| Kum14a | Kumistavi | Georgia | 2012 | MK031059 | MK031121 | This study |
| Kum16 | Kumistavi | Georgia | 2012 | MK031060 | MK031122 | This study |
| Kum19 | Kumistavi | Georgia | 2012 | MK031061 | MK031164 | This study |
| Kum20 | Kumistavi | Georgia | 2012 | MK031062 | MK031123 | This study |
| Kum37 | Kumistavi | Georgia | 2012 | MK031063 | MK031124 | This study |
| Kum39 | Kumistavi | Georgia | 2012 | MK031064 | MK031125 | This study |
| Kum40 | Kumistavi | Georgia | 2012 | MK031065 | MK031126 | This study |
| Kum48 | Kumistavi | Georgia | 2012 | MK031066 | MK031127 | This study |
| Muk37b | Mukhuri | Georgia | 2010 | MK031067 | MK031162 | This study |
| Sab3 | Satsable | Georgia | 2012 | MK031068 | MK031128 | This study |
| Sab4 | Satsable | Georgia | 2012 | MK031069 | MK031129 | This study |
| Sab5 | Satsable | Georgia | 2012 | MK031070 | MK031130 | This study |
| Sab16 | Satsable | Georgia | 2012 | MK031071 | MK031163 | This study |
| Sab19 | Satsable | Georgia | 2012 | MK031072 | MK031131 | This study |
| Sab32 | Satsable | Georgia | 2012 | MK031073 | MK031132 | This study |
| Sab37 | Satsable | Georgia | 2012 | MK031074 | MK031133 | This study |
| Sab41 | Satsable | Georgia | 2012 | MK031075 | MK031134 | This study |
| Sab48a | Satsable | Georgia | 2012 | MK031076 | MK031135 | This study |
| Sab48b | Satsable | Georgia | 2012 | MK031077 | MK031136 | This study |
| Sab50 | Satsable | Georgia | 2012 | MK031078 | MK031165 | This study |
| SacB2 | Satsire | Georgia | 2010 | MK031079 | MK031137 | This study |
| SacB10 | Satsire | Georgia | 2010 | MK031080 | MK031138 | This study |
| Sat20 | Satsire | Georgia | 2012 | MK031081 | MK031139 | This study |
| She2 | Shemoqmedi | Georgia | 2012 | MK031082 | MK031140 | This study |
| She6 | Shemoqmedi | Georgia | 2012 | MK031083 | MK031141 | This study |
| She9 | Shemoqmedi | Georgia | 2012 | MK031084 | MK031142 | This study |
| She12 | Shemoqmedi | Georgia | 2012 | MK031085 | MK031143 | This study |
| She24 | Shemoqmedi | Georgia | 2012 | MK031086 | MK031144 | This study |
| She27 | Shemoqmedi | Georgia | 2012 | MK031087 | MK031145 | This study |
| She30 | Shemoqmedi | Georgia | 2012 | MK031088 | MK031146 | This study |
| She31 | Shemoqmedi | Georgia | 2012 | MK031089 | MK031147 | This study |
| She35 | Shemoqmedi | Georgia | 2012 | MK031090 | MK031148 | This study |
| She37a | Shemoqmedi | Georgia | 2012 | MK031091 | MK031149 | This study |
| She38 | Shemoqmedi | Georgia | 2012 | MK031092 | MK031150 | This study |
| She40 | Shemoqmedi | Georgia | 2012 | MK031093 | MK031166 | This study |
| She46 | Shemoqmedi | Georgia | 2012 | MK031094 | MK031151 | This study |
| She49 | Shemoqmedi | Georgia | 2012 | MK031095 | MK031152 | This study |
| Tal14a | Taleri | Georgia | 2011 | MK031096 | MK031153 | This study |
| Tal24a | Taleri | Georgia | 2011 | MK031097 | no sequence | This study |
| Tkhi23 | Tkilnari | Georgia | 2011 | MK031098 | MK031154 | This study |
| Tkhi35b | Tkilnari | Georgia | 2011 | MK031099 | MK031155 | This study |
| Tkhi39a | Tkilnari | Georgia | 2011 | MK031100 | MK031159 | This study |
| Tkhi48 | Tkilnari | Georgia | 2011 | MK031101 | MK031167 | This study |
| Thki50 | Tkilnari | Georgia | 2011 | MK031102 | MK031156 | This study |
| Tska11 | Tskalthashua | Georgia | 2011 | MK031103 | MK031157 | This study |
| Tska17b | Tskalthashua | Georgia | 2011 | MK031104 | MK031160 | This study |
| Tska28 | Tskalthashua | Georgia | 2011 | MK031105 | MK031158 | This study |
| Tska34b | Tskalthashua | Georgia | 2011 | MK031106 | MK031161 | This study |
|  |  |  |  |  |  |  |
| **CHV-1 reference strains** | |  |  |  |  |  |
| EP713_subtypeF1 |  | France |  | M57938.1 (full genome) | | Shapira et al. [1] |
| Euro7_subtypeI |  | Italy |  | AF082191.1 (full genome) | | Chen and Nuss [2] |
| EP721_subtypeI |  | Italy |  | DQ861913.1 (full genome) | | Lin et al. [3] |
| M2021_subtypeF2 |  | France |  | MF421718 (full genome) | | Mlinarec et al. [4] |
| M1728_subtypeF2 |  | France |  | MF421717 (full genome) | | Mlinarec et al. [4] |
| M1372_subtypeD |  | Germany |  | KF914717.1 | MK069425 | Peters et al. [5]; this study |
| M1147_subtypeE |  | Spain |  | MK069427 | MK069426 | This study |
| AS326_subtypeD |  | Spain |  | KY002100.1 | KY002082.1 | Trapiello et al. [6] |
| AS328_subtypeD |  | Spain |  | KY002102.1 | KY002083.1 | Trapiello et al. [6] |

**References for Table S1**

1. Shapira, R.; Choi, G.H.; Nuss, D.L. Virus-like genetic organization and expression strategy for a double-stranded-RNA genetic element associated with biological control of chestnut blight. *Embo J* **1991**, *10*, 731-739.

2. Chen, B.S.; Nuss, D.L. Infectious cDNA clone of hypovirus CHV1-Euro7: a comparative virology approach to investigate virus-mediated hypovirulence of the chestnut blight fungus *Cryphonectria parasitica*. *J Virol* **1999**, *73*, 985-992.

3. Lin, H.; Lan, X.; Liao, H.; Parsley, T.B.; Nuss, D.L.; Chen, B. Genome sequence, full-length infectious cDNA clone, and mapping of viral double-stranded RNA accumulation determinant of hypovirus CHV1-EP721. *J Virol* **2007**, *81*, 1813-1820, doi:10.1128/JVI.01625-06.

4. Mlinarec, J.; Nuskern, L.; Jezic, M.; Rigling, D.; Curkovic-Perica, M. Molecular evolution and invasion pattern of *Cryphonectria hypovirus 1* in Europe: Mutation rate, and selection pressure differ between genome domains. *Virology* **2018**, *514*, 156-164, doi:10.1016/j.virol.2017.11.011.

5. Peters, F.S.; Busskamp, J.; Prospero, S.; Rigling, D.; Metzler, B. Genetic diversification of the chestnut blight fungus *Cryphonectria parasitica* and its associated hypovirus in Germany. *Fungal Biol* **2014**, *118*, 193-210, doi:10.1016/j.funbio.2013.11.009.

6. Trapiello, E.; Rigling, D.; Gonzalez, A.J. Occurrence of hypovirus-infected *Cryphonectria parasitica* isolates in northern Spain: an encouraging situation for biological control of chestnut blight in Asturian forests. *Eur J Plant Pathol* **2017**, *149*, 503-514, doi:10.1007/s10658-017-1199-4.
